# Identifying Key Somatic Copy Number Alterations Driving Dysregulation of Cancer Hallmarks in Lower-Grade Glioma

**DOI:** 10.3389/fgene.2021.654736

**Published:** 2021-06-07

**Authors:** Yao Zhou, Shuai Wang, Haoteng Yan, Bo Pang, Xinxin Zhang, Lin Pang, Yihan Wang, Jinyuan Xu, Jing Hu, Yujia Lan, Yanyan Ping

**Affiliations:** College of Bioinformatics Science and Technology, Harbin Medical University, Harbin, China

**Keywords:** somatic copy number alteration, driver genes, random walk with restart, cancer hallmark, LGG, regression analysis

## Abstract

Somatic copy-number alterations (SCNAs) are major contributors to cancer development that are pervasive and highly heterogeneous in human cancers. However, the driver roles of SCNAs in cancer are insufficiently characterized. We combined network propagation and linear regression models to design an integrative strategy to identify driver SCNAs and dissect the functional roles of SCNAs by integrating profiles of copy number and gene expression in lower-grade glioma (LGG). We applied our strategy to 511 LGG patients and identified 98 driver genes that dysregulated 29 cancer hallmark signatures, forming 143 active gene-hallmark pairs. We found that these active gene-hallmark pairs could stratify LGG patients into four subtypes with significantly different survival times. The two new subtypes with similar poorest prognoses were driven by two different gene sets (one including *EGFR, CDKN2A, CDKN2B, INFA8*, and *INFA5*, and the other including *CDK4, AVIL*, and *DTX3*), respectively. The SCNAs of the two gene sets could disorder the same cancer hallmark signature in a mutually exclusive manner (including E2F_TARGETS and G2M_CHECKPOINT). Compared with previous methods, our strategy could not only capture the known cancer genes and directly dissect the functional roles of their SCNAs in LGG, but also discover the functions of new driver genes in LGG, such as *IFNA5, IFNA8*, and *DTX3*. Additionally, our method can be applied to a variety of cancer types to explore the pathogenesis of driver SCNAs and improve the treatment and diagnosis of cancer.

## Introduction

Lower-grade glioma is the most common malignant primary tumor associated with a wide range of survival times from 1 to 15 years (Ostrom et al., 2013; Tan et al., 2020; Wu et al., 2020). This tumor is made up of World Health Organization (WHO) grade II and grade III gliomas, showing slower disease progress compared with glioblastoma (GBM, grade IV) (Ceccarelli et al., 2016). Until recently, comprehensive treatments of lower-grade glioma (LGG) have mainly focused on neurosurgical resection, radiotherapy, and chemotherapy, but tumor recurrence and drug resistance are inevitable due to their highly invasive nature and extensive genetic heterogeneity (Ceccarelli et al., 2016; Hayes et al., 2018). Therefore, it is urgent to better understand the pathogenesis and molecular characteristics of LGG and develop more driver genes for the early diagnosis and prognostic prediction of LGG.

Somatic copy-number alteration (SCNA), as a crucial somatic genetic alteration event, is exceedingly common in cancer (Baudis, 2007; Lopez-Gines et al., 2010; Negrini et al., 2010; Hollander et al., 2011). Up to 15% of the human genome is located in the CNV area (Stankiewicz and Lupski, 2010). Analysis of copy number profiles from multiple cancers shows that each sample carries an average of 24 amplifications and 18 deletions, and even some cancer samples have an average of 17% amplifications and 16% deletions in the human genome, compared with an average of 0.35%, less than 0.1% in normal samples (Beroukhim et al., 2010). However, the extensive complexity and high degree of heterogeneity of the human cancer genome have posed challenges in identifying these key genes driving the initiation and progression of LGG (Tamborero et al., 2013; Alizadeh et al., 2015; Yadav and De, 2015). Hence, identifying the key driver SCNAs that play causal roles in oncogenesis is crucial for understanding the occurrence and development of the LGG.

Recently, many approaches have been proposed to identify driver SCNAs. The traditional method identified the drivers by executing functional experiments on the genes located in the region (Pon and Marra, 2015). For example, through functional tests, Hagerstrand et al. (2013) found that *SKIL* and *TLOC1* that frequently amplified in multiple cancers were identified as drivers of 3q26, leading to subcutaneous tumor growth. However, the traditional method was time-consuming and expensive. With the accumulation of multi-dimensional omic data, multiple computational algorithms for identifying driver SCNAs have emerged. Some computational algorithms discovered drivers based on the alteration frequencies in cancer populations. For instance, GISTIC detected significant SCNAs by calculating the significance of gene amplification or deletion across cancer samples (Mermel et al., 2011). The effects of copy number alterations on the expressions of other genes were introduced to reflect the functional influence of driver SCNAs. DriverNet is a computational framework to identify the minimum number of driver genes that explain transcriptome changes with the largest extent across cancer samples (Bashashati et al., 2012). Akavia et al. (2010) identified driver genes with higher frequencies by regulating expression to influence the expressions of other gene sets by integrating copy number and expression profiles in human cancers. However, it was extremely limited in making clear the functions of SCNAs in cancer and how they contributed to malignant phenotypes.

Random walk with restart (RWR) (Kohler et al., 2008), as one of the classic network propagation algorithms, can capture the global structure of the network and has the characteristics of robustness to the noise in the network (Zhang C. et al., 2015). In the field of biology, RWR has been used to capture the disordered information that disease-related genes transmit through the topological structures of biological networks for identifying disease genes, mining disease modules, and predicting drug target (Robinson et al., 2008; Li and Patra, 2010; Cowen et al., 2017). HotNet2 and Hierarchical HotNet applied a similar RWR procedure but implemented different approaches to identify disease-related modules (Leiserson et al., 2015; Reyna et al., 2018). These studies indicate that the RWR algorithm could help us to capture the driver effects of SCNAs through topological structures of biological networks.

The present study developed an integrated computational framework to identify the key SCNAs driving the dysregulation of cancer hallmarks in LGG. We used the RWR algorithm to build the candidate gene-hallmark network by estimating the driver effects of seed genes on the cancer hallmarks based on the weighted co-expression protein interaction network. We also used linear regression analysis to identify the driver gene sets that cooperatively contributed to the dysregulation of hallmarks. We found that driver gene-hallmark pairs could identify two new LGG subtypes with a similarly poor prognosis.

## Materials and Methods

The copy number and mRNA expression data of LGG patients were obtained from the cBioPortal database^1^. Additionally, high confidence protein interaction data were obtained from STRING (Franceschini et al., 2013) which included 419,720 pairs of interactions involving 17,155 protein coding genes (PCGs). Finally, we downloaded 50 hallmark signature gensets from the Msigdb database^2^.

### Overview of the Method for Identifying the Key Copy Number Alterations Driving the Dysregulation of Cancer Hallmarks

**FIGURE 1:**
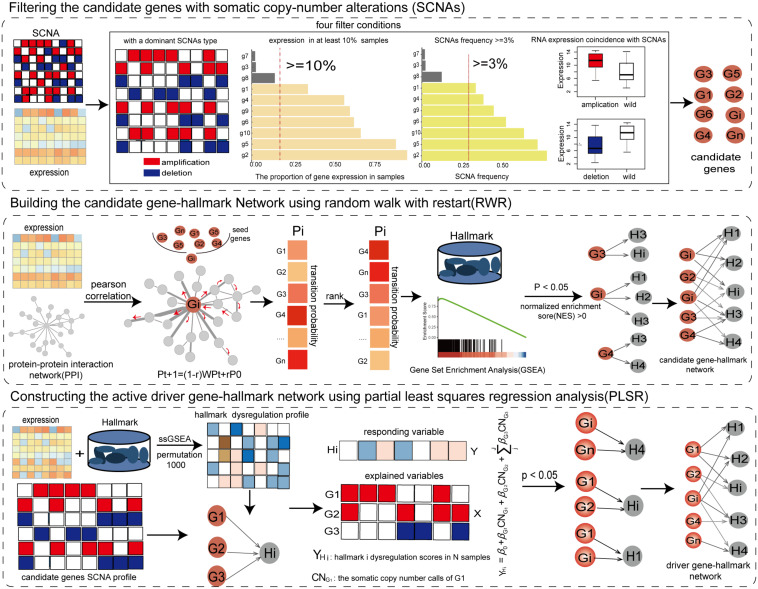
The workflow of the integrative method for identifying SCNAs driving the dysregulation of cancer hallmarks.

We developed an integrated method to identify key SCNAs using network propagation algorithm and regression analysis through integrating the profiles of copy number alterations and gene expression and weighted co-expression protein interaction network (Figure 1).

### Filtering the Candidate Genes With Somatic Copy-Number Alterations

Genes with SCNAs in the copy number profile were filtered as candidate genes by integrating profiles of copy number and gene expression if these genes met the following four criteria: (1) Genes should have a dominant SCNAs type (either high-level amplification or homogenous deletion) using the binomial test at *P* < 0.05 (Zhang et al., 2017). (2) Genes should be expressed in at least 10% of cancer samples (Zhou et al., 2017). (3) Genes with the dominant type should be altered in at least 3% of cancer samples. (4) The dominant SCNAs of genes should have a concordant influence on their expression (one-tailed Wilcoxon rank-sum test with FDR < 0.05). The genes selected by the four criteria were used to identify driver genes.

### Building the Candidate Gene-Hallmark Network Using Random Walk With Restart

By integrating the gene expression profile of cancer samples and protein interaction network, the weighted co-expression protein interaction network was constructed by calculating the expression correlation value for each pair of protein interactions. The weighted protein interaction network was unsigned, in which the weight of each edge represented the active extent between PCGs. According to the RWR principle, the higher the correlation coefficient between PCGs, the greater the probability of transmitting the imbalanced information. To measure the driver influence of candidate genes, each candidate gene was used as seed node to carry out a RWR (Kohler et al., 2008) on the weighted co-expression protein interaction network. The stable transfer probabilities from the seed gene reflected the driver extent by the seed gene on the genes in the protein interaction network. The information flow could restart from the seed genes with probability *r*.

$$P_{t\,1}=\left(1-r\right)\,W\,P_{t}+r\,P_{0}$$

where *r* was set to 0.3; P0 were the initial probabilities of genes, in which the probabilities of seed genes were 1; Pt were the transfer probabilities of genes at the t step; The Pt + 1 characterized the stable transfer probabilities; *W* was the normalized transfer matrix of the weighted co-expression protein interaction network; the random walk process reached the steady-state when the maximum difference between Pt + 1 and Pt was less than <1e−8. Based on the stable transfer probabilities, we used gene set enrichment analysis (GSEA) (Subramanian et al., 2005) to identify the significant cancer hallmarks driven by the candidate gene at NES (normalized enrichment score) >0 and *P* < 0.05. The candidate gene and its driving cancer hallmarks formed candidate gene-hallmark pairs and were then used to build a candidate gene-hallmark network by collecting all candidate gene-hallmark pairs from all candidate genes.

### Constructing the Active Driver Gene-Hallmark Network Using Partial Least Squares Regression Analysis

The gene expression profile was standardized by dividing the mean expression to eliminate the natural expression levels of genes in cancer samples. To assess dysfunctional activities of hallmark signatures in each sample, we built the significant dysfunctional profile of cancer hallmark signatures using single-sample GSEA (ssGSEA) (Hanzelmann et al., 2013). To measure the significance of dysfunctional activity, we permuted the gene expression profile of each sample 1,000 times, and recalculated 1,000 random scores of dysfunctional activities for each hallmark signature. The significance of activation was calculated as the frequency with which the random activity differences were greater than the actual activity score. Conversely, the significance of inactivation was calculated by the frequency of the random activity differences less than the real one. The significant activation/inactivation hallmark signatures in each sample were identified at FDR < 0.05.

To further determine the driver genes contributing to the variation of dysfunctional activities of hallmarks, a linear regression model was used to dissect the driver extents of driver genes on hallmarks based on driver relationships from the candidate gene-hallmark network. For each significant dysfunction hallmark in cancer samples, the candidate genes driving this hallmark were extracted. The partial least squares regression (PLSR) model was adopted to identify driver genes that cooperatively contributed to the dysfunctional activities of this hallmark (Bjornstad et al., 2004), in which the significant activity scores of the hallmark in cancer samples were the responding variables (Y), and the SCNAs statuses of candidate genes were explained variables (X).

The significance of the driver effects of candidate genes on dysfunctional activities of the hallmark was assessed using 10-fold cross-validation in R package “pls” (R functions of PLSR, RMSEP, and Jack.test). Subsequently, the driver genes that significantly contributed to the dysfunction of the hallmark with significant coefficients (*P* < 0.05) were identified, and active driver gene-hallmark pairs were formed. The active driver gene-hallmark network was constructed which consisted of all active driver gene-hallmark pairs. The genes in the active gene-hallmark network were identified as driver genes.

## Results

### Identifying Cancer Hallmarks Driven by Somatic Copy Number Alterations Using Random Walk With Restart in LGG

In total, 511 LGG samples detected in both expression profile and copy number profile were used for subsequent analysis. Among the 20,530 PCGs (protein coding genes) with SCNAs, 19,059 PCGs were expressed in at least 10% of LGG samples. 10,280 PCGs showed one dominant SCNAs type (high-level amplification or homozygous deletion) (binomial test, *P* < 0.05), of which 1,736 PCGs somatic copy number alterations significantly affected their expressions (FDR < 0.05). Eventually, 391 candidate PCGs with SCNAs frequency greater than 3% were identified, and only 40% of cancer samples occurred SCNAs of these candidate PCGs.

The weighted protein interaction network was constructed in LGG, in which each interaction was weighted by the expression correlation coefficient of the gene pair, representing the active extent of the interaction. The dysfunctional information of genes could be diffused following the topological structure of the weighted protein interaction network. The genes with higher expression correlation coefficients had higher probabilities to receive dysfunctional information. To estimate the driver effects of candidate genes on cancer hallmarks, each candidate gene was sowed as a seed node in the weighted protein interaction network, and the RWR algorithm was used to calculate the stable transition probability from the candidate gene to the other genes in the protein interaction network. The significant cancer hallmarks driven by the candidate gene were identified using GSEA (Subramanian et al., 2005) according to the rank of stable transition probabilities (NES > 0, *P* < 0.05). For instance, *EGFR* amplification was frequent in LGG, which significantly upregulated the *EGFR* expression (Supplementary Figure 1). We found that *EGFR* amplification significantly affected many cancer hallmarks, including development signature (EPITHELIAL_MESENCHYMAL_TRANSITION, *P* = 0.012), immune signature (ALLOGRAFT_REJECTION, *P* = 0.004; IL6_JAK_STAT3_SIGNALING, *P* = 0.005; INTERFERON_ GAMMA_ RESPONSE, *P* = 0.013) (Supplementary Figure 2A).

**FIGURE 2:**
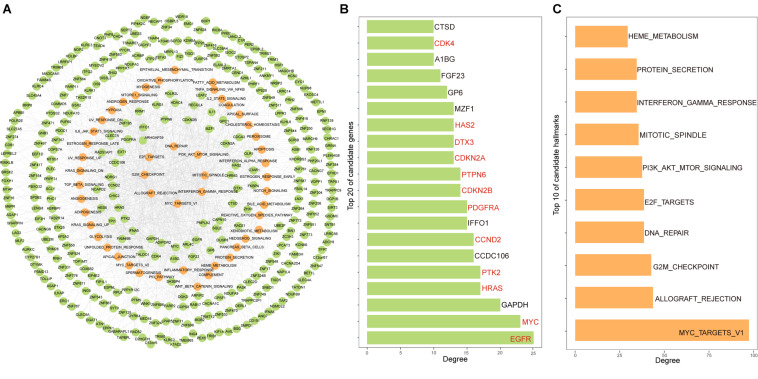
Constructing the candidate gene-hallmark network using random walk for LGG. **(A)** The candidate gene-hallmark network, the green dots represent candidate genes, and the yellow ones represent hallmarks signature affected by candidate genes; **(B)** the top 20 genes with a high contribution degree of the candidate gene-hallmark network, the genes marked in red represent known cancer genes; **(C)** the top 10 hallmarks with a high contribution degree of the candidate gene-hallmark network.

By assembling cancer hallmarks driven by all candidate genes, the candidate gene-hallmark network was constructed, in which nodes represented candidate genes and cancer hallmarks, and edges represented driver relationships among them. The candidate gene-hallmark network involved 1,177 gene-hallmark pairs including 329 PCGs and 50 hallmarks (Figure 2A). We ranked candidate genes in descending order according to their degree in the gene-hallmark network and found that 60% of the top 20 genes were known cancer genes including *EGFR*, *MYC, HRAS, PTK2, CCND2, PDGFRA, CDKN2B, PTPN6, CDKN2A, DTX3, HAS2*, and *CDK4* (Figure 2B). For ranking hallmark signatures, among the top ten hallmarks, four were related to proliferation signatures, two were related to immune signatures, one was related to signal signature, one was related to pathway signature, one was related to DNA damage signature, and one was related to the metabolic signature (Figure 2C).

### The Driver SCNAs in LGG Based on the Active Gene-Hallmark Driving Network

**FIGURE 3:**
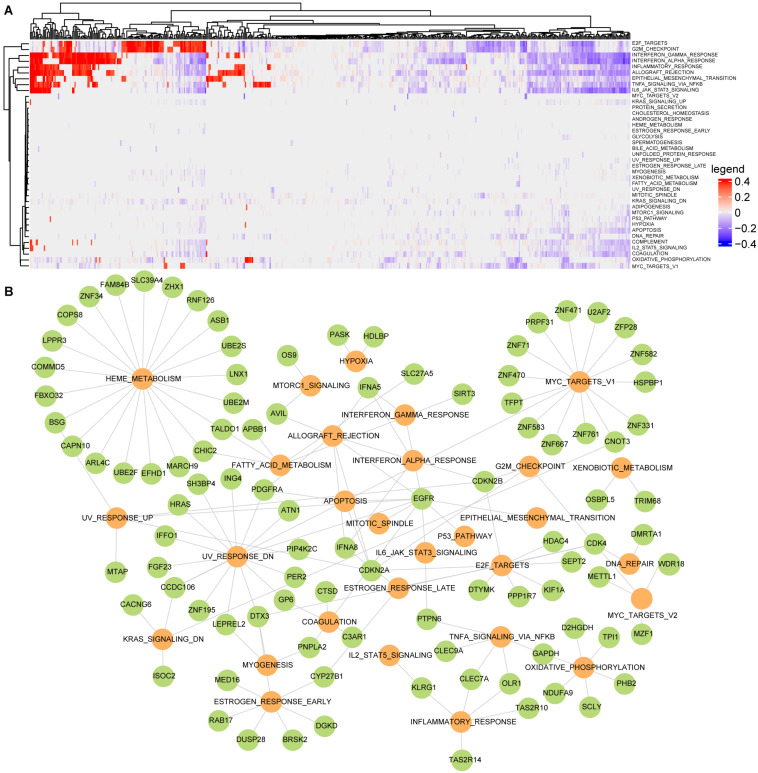
Building the active driver gene-hallmark network using regression analysis for LGG. **(A)** The dysfunctional profile of cancer hallmarks for LGG; **(B)** the active gene-hallmark network, the green dots represent driver genes, and the yellow ones represent disordered hallmark signatures.

Single-sample gene set enrichment analysis algorithm was used to evaluate the active scores of cancer hallmarks for each LGG patient. In order to characterize dysfunctional activities of hallmark signatures in each patient, the dysfunctional profile of cancer hallmarks was constructed by permutating the expression profile of each sample 1,000 times at the threshold of FDR < 0.05 (Figure 3A). A total of 39 cancer hallmarks were dysregulated in at least one LGG patient. Among them, eight hallmarks were dysregulated in more than 50% of LGG samples including two proliferation signatures (E2F_TARGETS and G2M_CHECKPOINT), four immune signatures (INTERFERON_ GAMMA_ RESPONSE, INTERFERON_ALPHA_RESPONSE, INFLAMMATORY_ RESPONSE, and ALLOGRAFT_ REJECTION), one development signature (EPITHELIAL_MESENCHYMAL_ TRANSITION), one signaling signature (TNFA_SIGNALING_ VIA_ NFKB). None of these cancer hallmarks showed a consistent state of activity in the LGG population. For example, the proliferation signature E2F_TARGETS was dysregulated in 339 LGG samples, which was significantly activated in 131 samples and inactivated in 208 samples. These results showed the activity heterogeneities of cancer hallmarks across the LGG population.

To further identify the driver factors underlying the activity heterogeneities of cancer hallmarks, the linear regression analysis was used to identify the driver gene sets that cooperatively contributed to activity changes of hallmarks by integrating the hallmark dysfunctional profile, the SCNAs profile of the candidate PCGs and the candidate gene-hallmark network. For each dysregulated cancer hallmark, the driver genes were identified at FDR < 0.05. The SCNAs of the driver genes could affect the activities of cancer hallmarks. For example, *EGFR* amplification significantly influenced the activities of EPITHELIAL_MESENCHYMAL_TRANSITION (*P* = 5.7e-09), ALLOGRAFT_ REJECTION (*P* = 7.2e-06), IL6_JAK_STAT3_SIGNALING (*P* = 0.002), INTERFERON_ GAMMA_ RESPONSE (*P* = 2.7e-08) (Supplementary Figure 2B). Finally, we found that 29 cancer hallmark signatures whose dysfunctional activities were significantly driven by 98 driver genes, forming an active gene-hallmark network with 143 gene-hallmark pairs (Figure 3B). Among 98 driver genes, 21 genes (including *CDKN2A, CDKN2B, FBXO32, SIRT3, ING4, PTPN6, CHIC2, RAD21, PDGFRA, EGFR, HRAS, ERC1, CDK4, HDLBP, DTX3, C3AR1, GPC1, TALDO1*, and *MTAP*) were recorded as cancer genes in at least one of four known databases [Cancer Gene Census in COSMIC (Sondka et al., 2018), TSGene (Zhao et al., 2016), Bushman^3^, and DriverDBv3 (Liu et al., 2020)]. Specifically, based on the degree of driver genes in the active gene-hallmark network, we found that 70% of the top 10 driver genes were cancer genes.

After conducting a manual literature search, we also found that 97.4% (75/77) of the remaining driver genes identified by our method were reported as cancer-associated genes *in vivo* or *in vitro* (Supplementary Table 1). For instance, Menezes et al. (2020) found that MTAP overexpression was associated with proliferation, migration, and invasion of glioma cells *in silico* and *in vitro* models. The low expression of PTPN6 was significantly associated with poor overall survival in bladder cancer patients and co-expression with TNFRSF14 (tumor necrosis factor receptor superfamily member 14) had a close correlation in breast cancer (Menezes et al., 2020). METTL1 promoted the proliferation and migration of hepatocellular carcinoma cells by inhibiting the PTEN signaling pathway and was associated with poor prognosis (Tian et al., 2019). Additionally, the ratio of known cancer genes in the active gene-hallmark network was elevated to 21.4% (21/98) by comparing that ratio of 15.5% (51/329) in the candidate gene-pathway network. These findings confirmed the driving roles of the identified PCGs.

### LGG Subtypes With Poor Prognosis Contributed by the Active Gene-Hallmark Network

**FIGURE 4:**
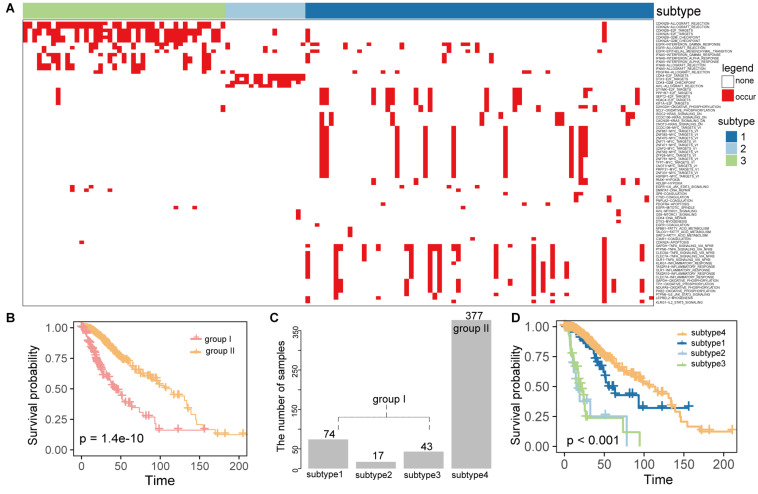
The gene-hallmark pairs identified were associated with LGG prognosis and characterize new subtypes. **(A)** The heatmap of gene-hallmark pairs contained 81 gene-hallmark pairs across 134 cancer samples for three subtypes; **(B)** comparison of survival time between the two groups with (group I) or without (group II) gene-hallmark pairs; **(C)** the bar plot showed the number of samples contained in each subtype; **(D)** the Kaplan–Meier analysis of subtypes identified by our method based on OS. *P*-value was calculated by the log-rank test among subtypes.

To investigate whether the gene-hallmark pairs from the active gene-hallmark driving network were associated with LGG prognosis and characterized new subtypes, we mapped the active gene-hallmark network into LGG patients and constructed a binary profile of gene-hallmark pairs for the LGG population, where each row was a pair of gene-hallmark, each column was a cancer sample and value referred to whether this gene-hallmark pair occurred in a certain sample. A gene-hallmark pair was considered to be present if the gene showed an SCNA and the cancer hallmark was significantly activated or inactivated in this sample. The binary profile of gene-hallmark pairs contained 81 gene-hallmark pairs across 511 cancer samples. We observed that 134 samples carried at least one gene-hallmark pair (group I) (Figure 4A) while the other 377 samples did not carry any of these pairs (group II), mainly because 303 samples out of 377 did not harbor any SCNAs. Using survival analysis, we found the survival time of 134 samples were significantly shorter than that of 377 samples (*P* = 1.4e-10, log-rank test, Figure 4B). Based on the active gene-hallmark pairs, these 134 patients (group I) were classified using consensus clustering [50 resamplings, 80% item resampling, partitioning around medoids (PAM) clustering method]. The consensus heatmap and cumulative distribution function were used to determine the optimal *K*. We identified three subtypes (1, 2, and 3) with the largest relative change in area under the CDF curve (*K* = 3) (Supplementary Figures 3A–C; Wilkerson and Hayes, 2010; Senbabaoglu et al., 2014). Finally, 511 LGG patients were stratified into four subtypes and the sample numbers of the four subtypes were 74, 17, 43, 377 accounting for 14.5, 3.3, 8.4, 73.8% of the 511 samples, respectively (Figure 4C). We compared the overall survival times of the three subtypes with that of subtype 4 (without gene-hallmark pairs), found that there were significant differences in overall survival time between the four groups (*P* < 0.001, log-rank test, Figure 4D). Subtype 2 and 3 showed significantly poorer prognosis than subtype 4 (*P* = 3.07e-13 for subtype 2; *P* = 0 for subtype 3, log-rank test, Supplementary Table 2), and subtype 1 showed shorter survival time with weaker significance. (*P* = 0.089, log-rank test, Supplementary Table 2). Furthermore, we compared the survival times among the three subtypes and found there were significant survival differences (*P* = 2.37e-07, log-rank test, Supplementary Table 2). The subtype 2 and 3 showed significantly shorter survival times than that of subtype 1 (*P* = 1.77e-05 for subtype 2; *P* = 1.64e-07 for subtype 3, log-rank test, Supplementary Table 2), but there was no significance in survival times between subtype 2 and 3(*P* = 0.89, log-rank test, Supplementary Table 2). Together, these results proved that the active gene-hallmark pairs could identify new LGG subtypes with poor prognoses.

#### The Subtype-Specific Gene-Hallmark Pairs in LGG

Subtypes 2 and 3 had the poorest prognosis and showed no significance in survival time. To explore the molecular mechanisms underlying these subtypes, we investigated the distribution of gene-hallmark pairs across LGG samples and identified the subtype specific gene-hallmark pairs using Fisher’s exact test at *P* < 0.05 for each subtype (Supplementary Table 3). For subtype 2, specific gene-hallmark pairs involving driver genes *CDK4, DTX3*, and *AVIL* were identified (Supplementary Figure 4A). For example, *CDK4* specifically and significantly drove two proliferation signatures (*P* = 6.02e-15 for E2F_TARGETS and *P* = 3.01e-12 for G2M_CHECKPOINT, Fisher’s exact test). Amplification of *CDK4, DTX3*, and *AVIL* significantly upregulated their gene expression (*P* = 2.4e-14 for *CDK4*, *P* = 1.7e-08 for *DTX3*, and *P* = 4.6e-10 for *AVIL*, Wilcoxon rank-sum test), and showed significant association with LGG poor prognosis (*P* = 1.2e-10 for *CDK4*, *P* = 8.03e-10 for *DTX3*, and *P* = 3.86e-6 for *AVIL*, log-rank test, Supplementary Figure 4B). *AVIL*, as a novel oncogene gene in LGG, was reported to play important roles in functions of cancer *in vivo* or *in vitro* through literature searches. For example, *AVIL* overexpression in GBM promoted cell proliferation and metastasis, leading to poor prognosis in patients (Xie et al., 2020). In the subtype 3, 12 specific gene-hallmark pairs were identified including driver genes (*EGFR, CDKN2A, CDKN2B, INFA8*, and *INFA5*), and their affected hallmarks were significantly presented in 43 LGG individuals (*P* < 0.05, Fisher’s exact test, Supplementary Figure 4A). *EGFR* amplification and the deletions of *CDKN2A, CDKN2B, INFA8*, and *INFA5* were also significantly associated with LGG poor prognosis (*P* < 0.001 for *CDKN2A, CDKN2B*, *P* = 1.42e-12 for *IFNA8*, *P* = 1.76e-12 for *IFNA5*, and *P* < 0.001 for *EGFR*, log-rank test, Supplementary Figure 4B).

Of note, we observed these specific gene-hallmark pairs in subtype 2 and 3, which showed obvious mutually exclusive patterns (Supplementary Figure 4A), but these driver genes drove the same dysregulated hallmark signatures such as proliferation (E2F_TARGETS and G2M_CHECKPOINT) and immunity signature (ALLOGRAFT_ REJECTION, INTERFERON_ GAMMA_ RESPONSE, and INTERFERON_ALPHA_ RESPONSE) in both subtype 2 and 3. These results implied that these two subtypes with the poorest prognosis were driven by different driver genes through dysregulating the same biological functions, further improving the potential of the identified active gene-hallmark pairs for identifying new LGG subtypes.

### Performance Evaluation of the Method

Known cancer genes were collected from multiple sources (Table 1). The driver genes identified by our method significantly overlapped with these known cancer gene sets (hypergeometric test, *P* < 0.05, Table 1). We then further compared our method with the other three previous methods, which also identified driver copy number alterations [Ping et al., 2020; Zhou et al., 2017, and DriverDBv3 (Liu et al., 2020)]. The results showed that our method could also significantly capture the driver genes identified by other methods with the Ping et al. (2020), Zhou et al. (2017) (hypergeometric test, *P* = 1.84e-05 for Ping et al. (2020), *P* = 4.23e-12 for Zhou et al. (2017), Table 1), but not significantly with DriverDBv3(hypergeometric test, *P* = 0.175, Table 1). Among them, Ping et al. (2020) could directly dissect long non-coding RNAs (lncRNAs) functional roles in cancers based on dysregulated ceRNA network induced by SCNAs but was limited in identifying potential driver SCNAs with low expression levels and SCNA frequencies, however, Zhou et al. (2017) and DriverDBv3 cannot dissect driver roles of genes in cancers in a direct manner (Supplementary Table 4). Additionally, the driver genes identified by our method also showed a significantly higher degree than the non-drivers whose expression levels were concordant with SCNAs in the PPI network (Supplementary Figure 5, *P* < 0.05, Wilcoxon rank-sum test). Our method could complement other methods, which identified novel driver genes contributing to cancer development. For instance, *DTX3* was a gene that had not been identified by other methods, but it had the ability to drive E2F_TARGETS hallmark signature dysfunction and was associated with LGG prognosis. These results suggest that our method is useful in identifying driver SCNAs that contribute to dysfunction cancer hallmarks in LGG.

**TABLE 1:** The performance of our method compared with six known cancer gene sources and three previous methods.

**Type**	**Cancer gene sets**	**Number of cancer genes**	**Number of gene intersections**	***P*-value**
Known cancer gene sets	CGC	723	10	0.00103
	TSG	638	7	0.0193
	Bushman	2,579	18	0.0237
	Tamborero et al., 2013	435	5	0.0385
	Rahman, 2014	114	6	9.29e-06
	An et al., 2016	1,571	16	0.0427
Previous method	Ping et al., 2020	37	4	1.84e-05
	Zhou et al., 2017	88	10	4.23e-12
	DriverDBv3	45	1	0.175

## Discussion

This article has proposed an integrative method to identify driver SCNAs and characterize the dysfunctional cancer hallmarks driven by these SCNAs in LGG. The active driver gene-hallmark pairs identified could stratify the LGG patients into four subtypes and identify two new subtypes with similar poor prognoses and with different underlying molecular mechanisms. We also discovered that different driver genes could disorder the same cancer hallmarks, leading to malignant phenotypes.

The dysfunctional effects of driver SCNAs were characterized by network propagation on the protein interaction networks. The selection of the specific forms of the protein interaction network was crucial. Our method used three forms of protein interaction network, including static protein interaction network, co-expression weighted PPI across only samples with SCNAs of the seed gene, weighted co-expression PPI across all cancer samples (our method). We found that the first two forms do not help to significantly capture the known cancer genes. For example, using the static PPI network, 147 driver PCGs dysregulating 27 hallmarks could not significantly overlap with TSGene (Zhao et al., 2016) (*P* = 0.225, hypergeometric test) and Bushman (*P* = 0.0622, hypergeometric test), which also did not account for the differences in co-expression patterns among genes from cancer to cancer. For the second, the identified active gene-hallmark network including 139 PCGs only weaker significantly overlapped with TSGene (Zhao et al., 2016) at the significance of *P* = 0.0926, which was not suitable for the PCGs with low SCNAs frequencies. The development of a comprehensive dynamic PPI network in the future could help us to better analyze the functional roles of SCNAs.

Two subtypes of poor prognosis were driven by different driver gene-hallmark pairs. The subtype 3 characterized by driver genes *CDKN2A* and *CDKN2B* was consistent with our previous studies (Ping et al., 2020), in which identified *CDKN2A* and *CDKN2B* could dysregulate the hallmark of G2M_CHECKPOINT based on miRNA-mediated ceRNA networks and further contributed to poor LGG prognosis. The SCNAs of *CDK4, AVIL*, and *DTX3* driven another new subtype with poor prognosis showed significant mutual exclusivity with that of *CDKN2A* and *CDKN2B* (Supplementary Figure 6). These phenomenons forming the hypothesis of functional redundancy were extensively used to identify the cancer genes (Sparks et al., 1998; Zhao et al., 2012; Babur et al., 2015). For example, Deng et al. (2018) identified cancer driver lncRNAs that were mutually exclusive with well-known driver genes based on functional redundancy hypothesis. Ping et al. (2014) showed that the glioma pathway was affected by 12 different genes (including *EGFR, PDGFRA, CAMK2B, AKT1, CDK4, MDM2, NRAS, PIK3CA, TGFA, SHC4, CDKN2A*, and *PDGFA*) with significant patterns of mutual exclusivity.

Another important finding of our method was that hallmark activity showed broad heterogeneity in the cancer population. For instance, proliferation (E2F_TARGETS and G2M_CHECKPOINT) and immunity signature (ALLOGRAFT_REJECTION) showed inconsistent activity statuses across the LGG population and dysregulated in more than 50% of LGG samples, but these hallmarks contributed malignant phenotypes driven by different genes in a mutually exclusive manner.

The four subtypes identified by our method were compared with WHO subtypes (*IDH* mutant and 1p/19q codeleted, *IDH* mutant and 1p/19q non-codeleted, *IDH* wild-type) (Louis et al., 2016). We found that subtype 2 and subtype 3 were mainly enriched in the IDH wild-type subtype, which showed the worst prognosis [58.8% (10/17) for subtype 2; 72.1% (31/43) for subtype3, Supplementary Figures 7A,B]. We identified the risk prognostic markers (including *CDKN2A*/*CDKN2B* deletion, *EGFR* amplification, *CDK4* amplification, IFNA5/IFNA8 deletion, *AVIL* amplification, and *DTX3* amplification) for *IDH* wild-type subtype from SCNAs level. For the *IDH* mutant and 1p/19q non-codeleted, and *IDH* wild-type subtypes, our subtypes could further divide them into four different subgroups with significant differences in survival times (*P* = 0.0045 for *IDH* mutant and 1p/19q non-codeleted, *P* < 0.0001 for *IDH* wild-type, log-rank test, Supplementary Figure 7C). Our subtypes were compared with the subtypes of LGG identified by eight different methods which had distinct molecular and clinical features (Ceccarelli et al., 2016). The comparison results were similar to that obtained from the comparison with WHO subtypes (Supplementary Figures 8–15). Our method provided the subtype and prognosis markers from the view of copy number alterations, which was served as an important complement for the existing methods.

To further verify our method, we applied our method to 507 lung adenocarcinoma (LUAD) samples and identified a total of 347 active gene-hallmark pairs containing 234 driver genes and 28 hallmarks. Based on the active gene-hallmark pairs, three subtypes were identified for LUAD samples, which contained 285, 170, 52 samples respectively (Supplementary Figure 16A). Survival analysis showed that there were significant differences in overall survival times among these three subtypes (*P* = 0.038, log-rank test, Supplementary Figure 16B). In the subtype 3 with the worst prognosis, specific gene-hallmark pairs involving driver genes *PAIP, RAP1B, AVIL, RAD1*, and *C1QTNF3* were identified. For example, amplification of *AVIL* specifically and significantly drove immunity signature (*P* < 0.05, for ALLOGRAFT_REJECTION, Fisher’s exact test). These results proved that our strategy has the potential to be extended to dissecting the driver roles of SCNAs in other cancer types.

It is noteworthy that this study captured a subset of novel driver genes including *IFNA5, IFNA8*, and *DTX3* with low SCNAs frequencies in LGG populations. Among these, *IFNA5* deletion (5.09% of LGG samples), *IFNA8* deletion (5.28% of LGG samples), and *DTX3* amplification (3.52% of LGG samples) indicated poor survival in LGG. Kao et al. (2018) found that the upregulation of *IFNA5* activated the ERβ-Ube3a interaction which further facilitated hepatic progenitor cell differentiation. Human epidemiologic studies showed that SNPs in *IFNA8* were significantly associated with the overall survival of patients with WHO grade 2 to 3 gliomas (Fujita et al., 2010). Byun et al. (2018) found that SNPs located in the promoter of *IFNA8* affected GBM patient prognosis. Ding et al. (2020) found that *DTX3* played an important role in the progression, and acted as an anti-oncogene in esophageal carcinoma. Gatza et al. (2014) discovered that *DTX3* was essential for cell proliferation in breast tumors.

In this work, each candidate PCG was sowed as a seed in the weighted protein interaction network to identify driver SCNAs using RWR, so our method was limited to identifying PCGs rather than non-coding genes and there was no way to identify seed PCGs that were not in the network. In the future, this problem may be solved with continuous improvement and expansion of the network or through different network forms, such as co-expression network, dysregulated ceRNA network. Additionally, Pearson correlation coefficient (PCC) was used to measure correlations between PCGs, which could not distinguish between direct and indirect relationships and ignored nonlinear relations between two genes due to only relying on the information of co-occurring events. Instead of PCC, we could in future use partial correlation similarly, using conditional mutual information to construct a direct association network (Zhang et al., 2013; Zhang X. et al., 2015; Liu et al., 2016).

The present study developed an integrative strategy to discover the key SCNAs driving dysfunction of cancer hallmarks and investigate the functional roles of driver SCNAs based on the weighted protein interaction network in the LGG population, which complimented previous methods. Our strategy could be extended to explore other driver factors, with the accumulation of multi-omics of multiple cancers.

## The Code for the Integrative Method

The code for the integrative method is available at https://github.com/zhouyao-max/Driver_SCNAs/tree/master/Driver_SCNAs.

## Data Availability Statement

The original contributions presented in the study are included in the article/Supplementary Material, further inquiries can be directed to the corresponding author/s.

## Author Contributions

YP, YL, and JH designed and guided this work. YZ, YP, and SW participated in data processing, program implementation, and manuscript writing. HY, BP, XZ, LP, YW, and JX contributed to data collecting and organized the figures and tables. All authors provided critical advice for the final manuscript.

## Conflict of Interest

The authors declare that the research was conducted in the absence of any commercial or financial relationships that could be construed as a potential conflict of interest.

## Footnotes

1 https://www.cbioportal.org/

2 https://www.gsea-msigdb.org/gsea/msigdb/

3 http://www.bushmanlab.org/links/genelists

## Abbreviations

LGG: lower-grade glioma
SCNAs: somatic copy-number alterations
RWR: random walk with restart
PCGs: protein coding genes
NES: normalized enrichment score
GSEA: gene set enrichment analysis
ssGSEA: single-sample gene set enrichment analysis.
