# Supplementary material for: Identifying Key Somatic Copy Number Alterations Driving Dysregulation of Cancer Hallmarks in Lower-Grade Glioma
Source: Front Genet. 2021 Jun 7;12:654736. doi: 10.3389/fgene.2021.654736 (PMC8215700; doi:10.3389/fgene.2021.654736)
Supplement: Supplementary file 1 [file Data_Sheet_1.docx]

**Supplemental Information**

**Identifying key somatic Copy-Number Alterations driving dysregulation of cancer hallmarks in Low-Grade Glioma**

**Supplement Figures**

Figure S1. SCNAs of *EGFR* significantly influenced their expression levels in LGG.

**
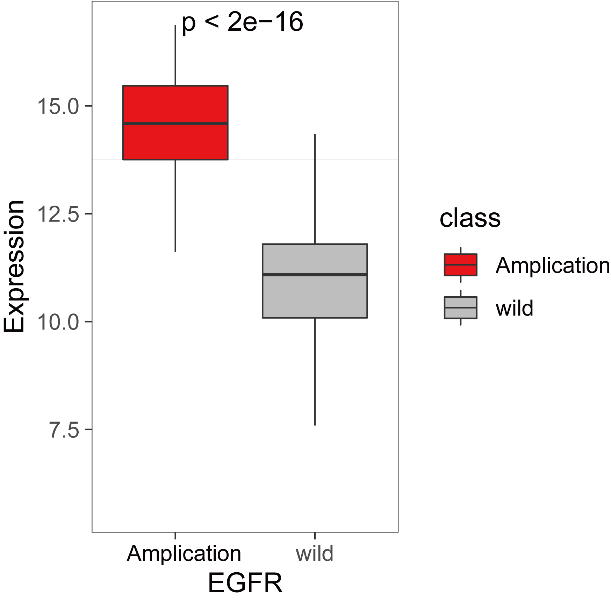
**

Figure S2. SCNAs of *EGFR* significantly influenced hallmarks in LGG. **(A)** Four hallmarks that were significantly affected by *EGFR* amplification were illustrated; **(B)** Four hallmarks activity score that were significantly affected by EGFR amplification were illustrated.


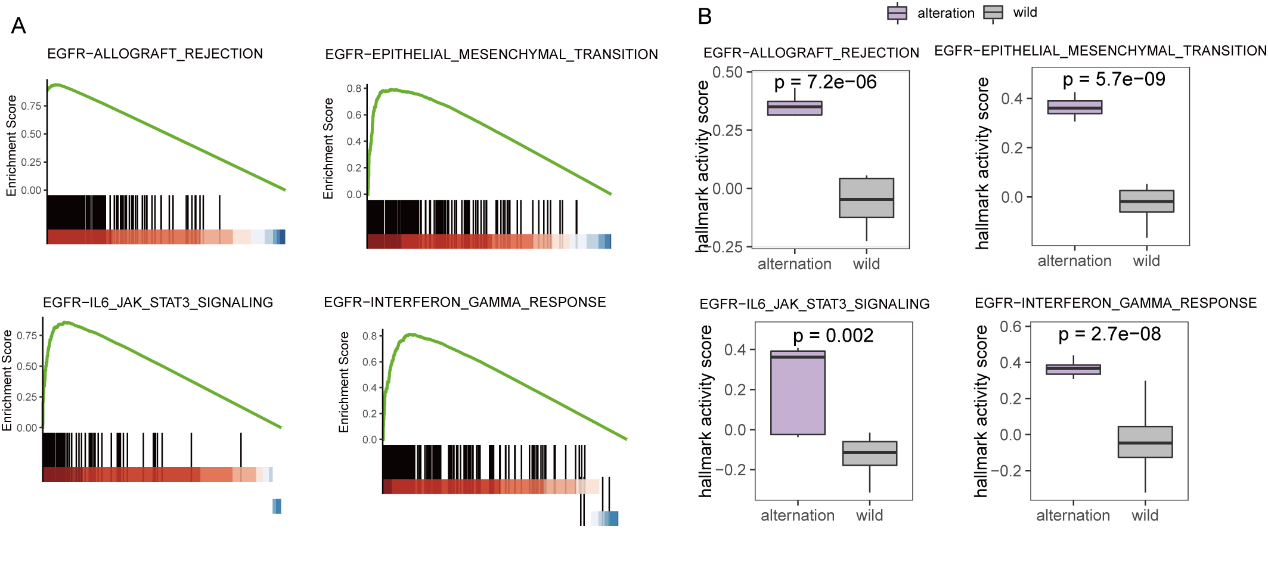


Figure S3. Consensus clustering based on active gene-hallmark pairs of LGG population in TCGA cohort **(A)** Consensus clustering cumulative distribution function (CDF) for k = 2–6; **(B)** Relative change in area under CDF curve for k = 2–6; **(C)** The consensus clustering heatmap for LGG.


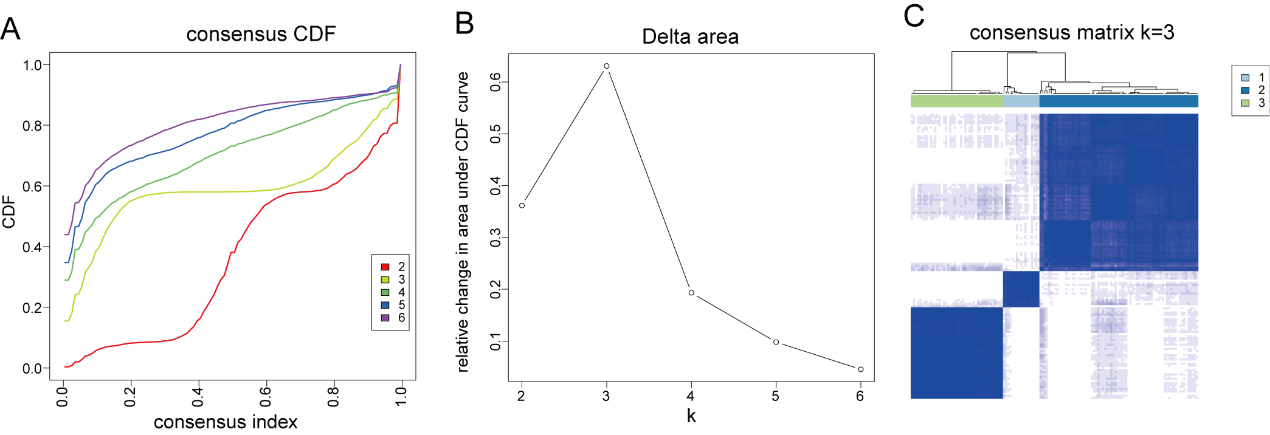


Figure S4. Characterize two new subtypes with poor prognosis; **(A)** Special gene-hallmark pairs for subtype 2 and 3; **(B)** Survival analysis of 8 driver genes (EGFR, CDKN2A, CDKN2B, INFA8, INFA5, CDK4, AVIL, DTX3) in LGG patients.


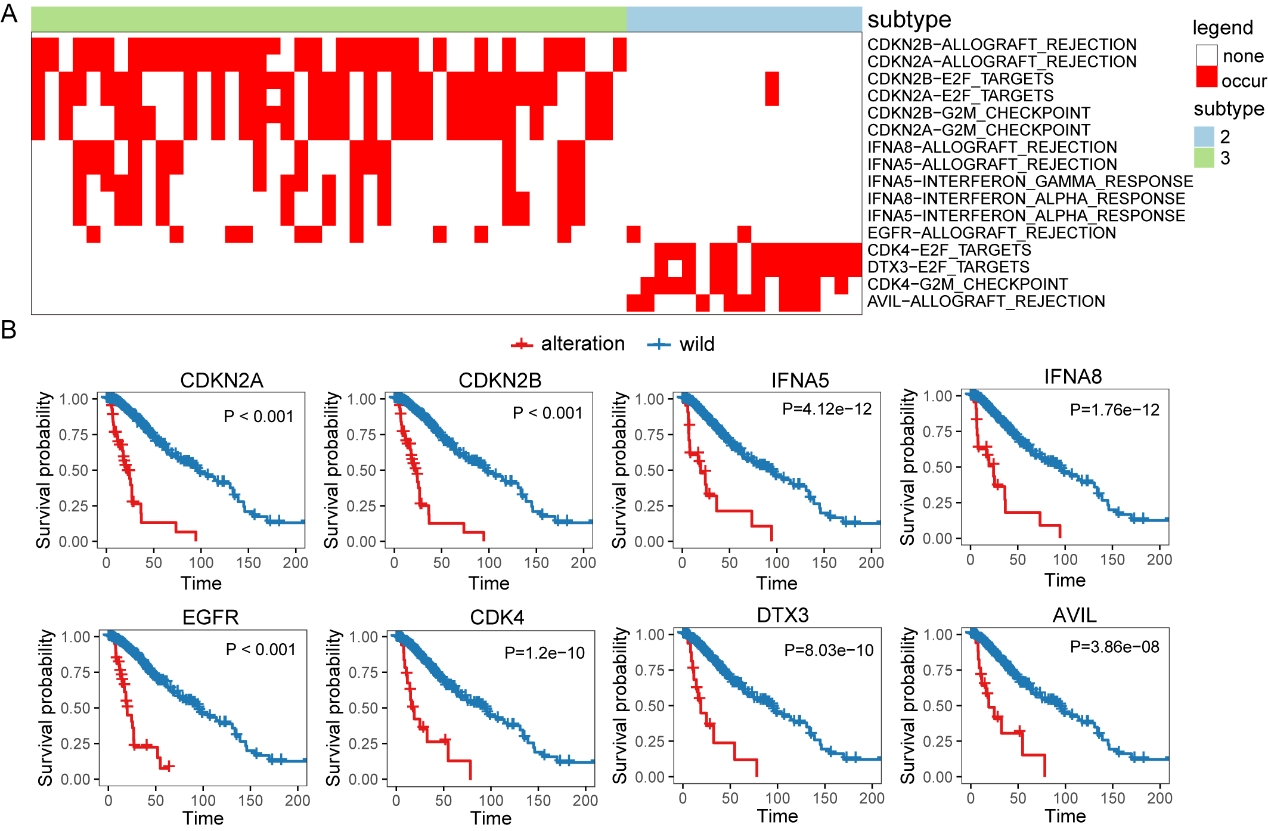


Figure S5. Comparison of degrees between drivers and non-drivers.


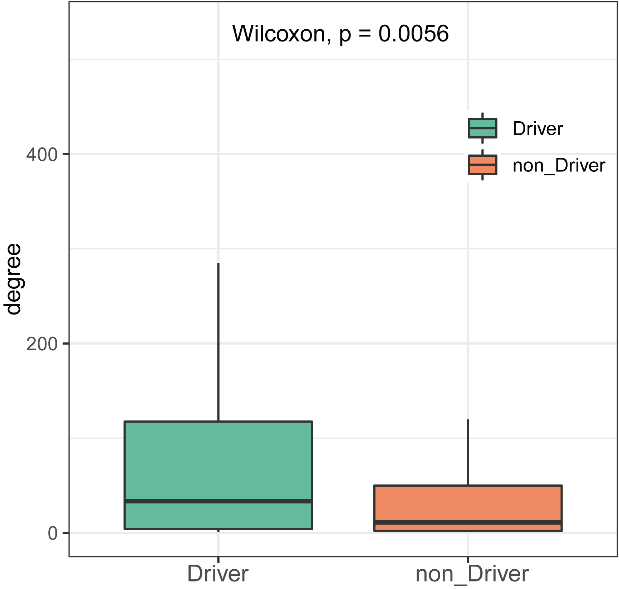


Figure S6. The SCNAs of CDK4, AVIL and DTX3 showed significant mutual exclusivity with that of CDKN2A/2B, IFNA5/8.


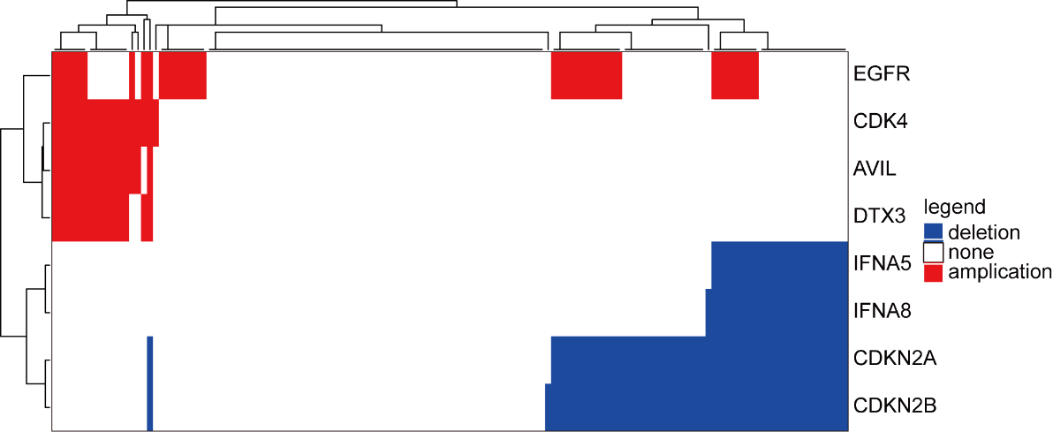


Figure S7. Comparison with WHO classification. **(A)** The Kaplan–Meier analysis of subtypes identified by other methods. P value was calculated by the log‐rank test among subtypes; **(B)** Bar plots showed the proportion of tumors stratified by WHO method within our subtypes; **(C)** The Kaplan–Meier analysis of each subtype which stratified according to our classification criteria. P value was calculated by the log‐rank test among subtypes.


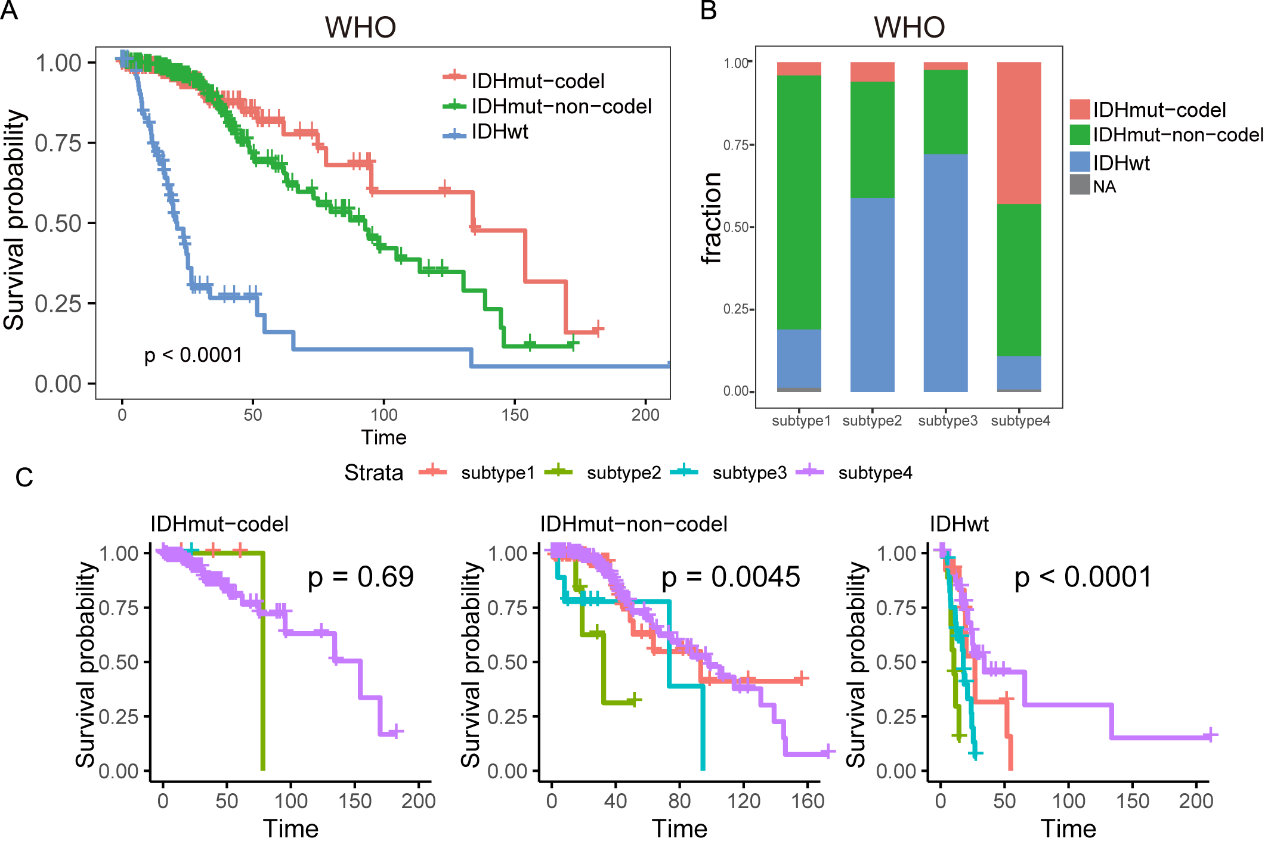


Figure S8. Comparison with other method(M1) **(A)** The Kaplan–Meier analysis of subtypes identified by M1. P value was calculated by the log‐rank test among subtypes; **(B)** Bar plots showed the proportion of tumors stratified by M1 within our subtypes; **(C)** The Kaplan–Meier analysis of each subtype which stratified according to our classification criteria. P value was calculated by the log‐rank test among subtypes; (CL, classical; ME, mesenchymal; NE, neural; PN, proneural).

**
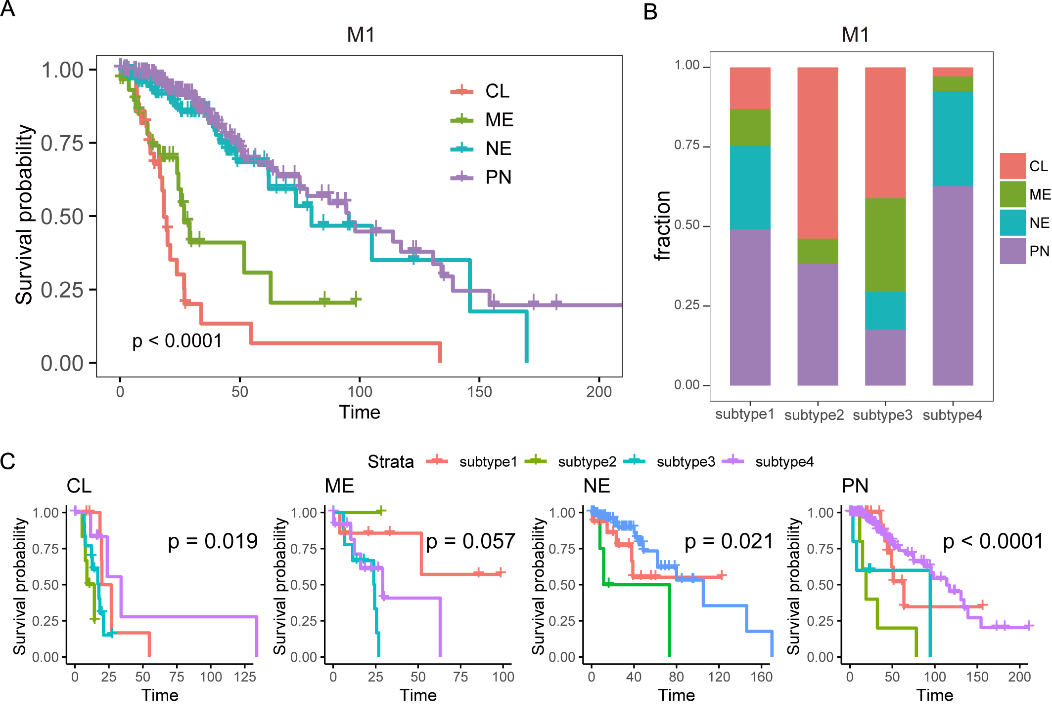
**

Figure S9. Comparison with other method(M2) **(A)** The Kaplan–Meier analysis of subtypes identified by M2. P value was calculated by the log‐rank test among subtypes; **(B)** Bar plots showed the proportion of tumors stratified by M2 within our subtypes; **(C)** The Kaplan–Meier analysis of each subtype which stratified according to our classification criteria. P value was calculated by the log‐rank test among subtypes.


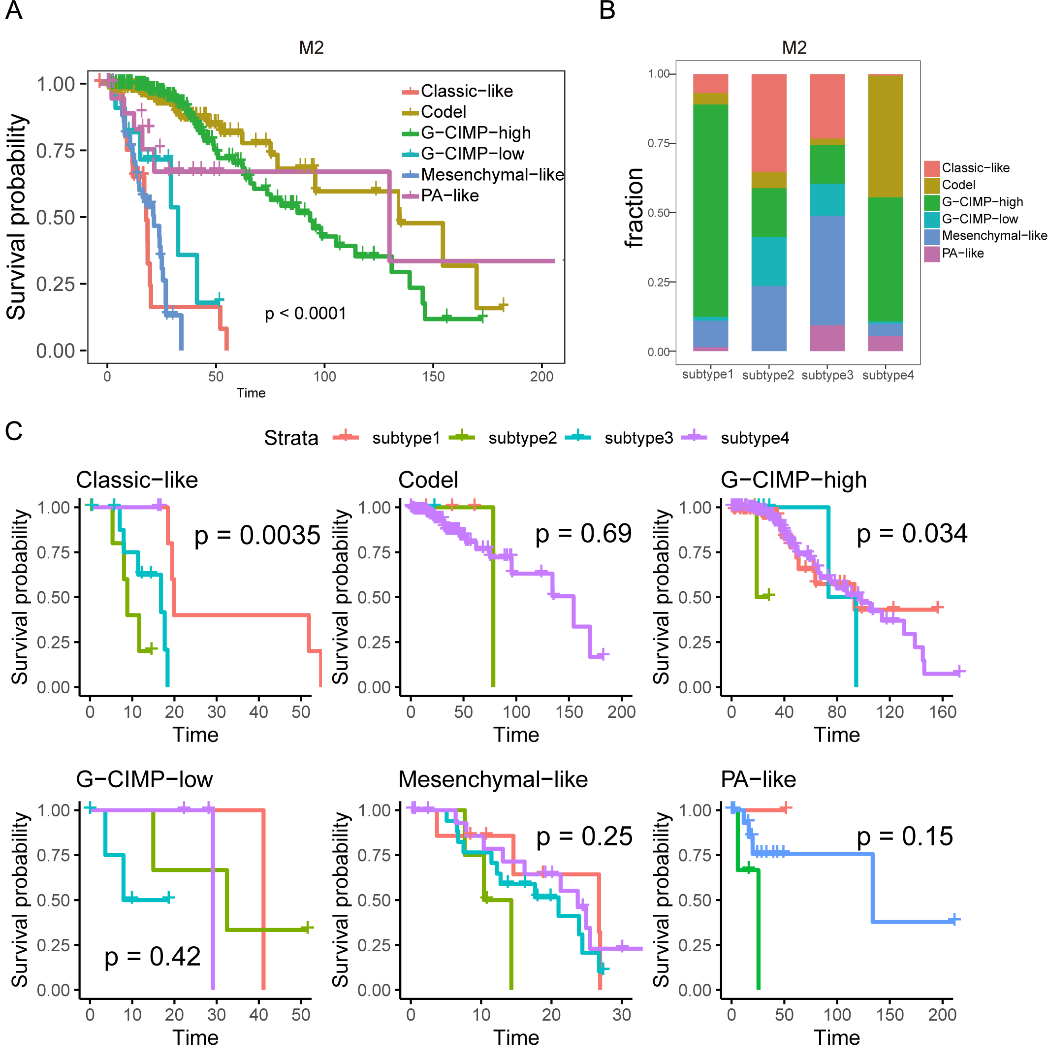


Figure S10. Comparison with other method(M3) **(A)** The Kaplan–Meier analysis of subtypes identified by M3. P value was calculated by the log‐rank test among subtypes; **(B)** Bar plots showed the proportion of tumors stratified by M3 within our subtypes; **(C)** The Kaplan–Meier analysis of each subtype which stratified according to our classification criteria. P value was calculated by the log‐rank test among subtypes.


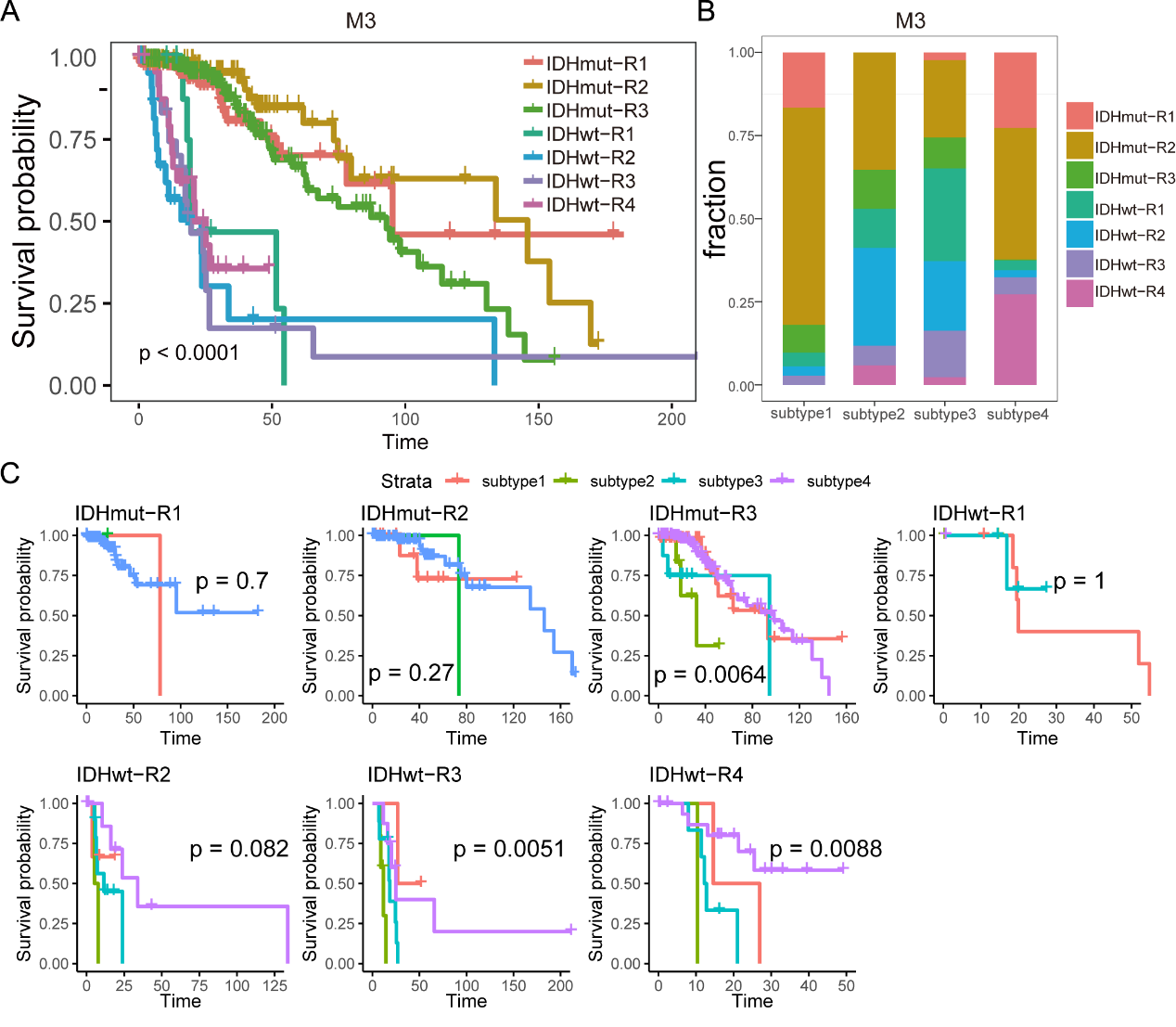


Figure S11. Comparison with other method(M4) **(A)** The Kaplan–Meier analysis of subtypes identified by M4. P value was calculated by the log‐rank test among subtypes; **(B)** Bar plots showed the proportion of tumors stratified by M4 within our subtypes; **(C)** The Kaplan–Meier analysis of each subtype which stratified according to our classification criteria. P value was calculated by the log‐rank test among subtypes.


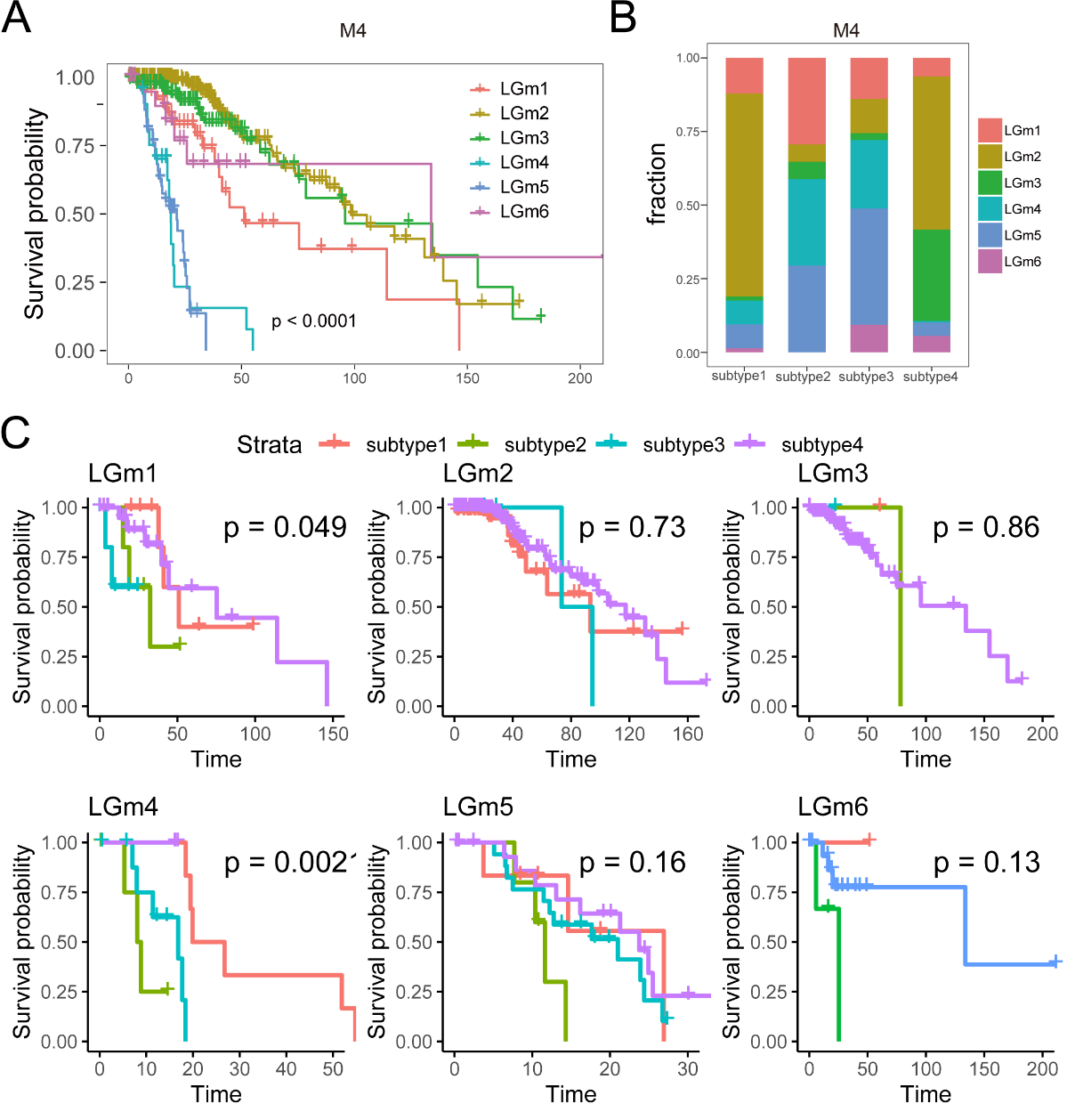


Figure S12. Comparison with other method(M5) **(A)** The Kaplan–Meier analysis of subtypes identified by M5. P value was calculated by the log‐rank test among subtypes; **(B)** Bar plots showed the proportion of tumors stratified by M5 within our subtypes; **(C)** The Kaplan–Meier analysis of each subtype which stratified according to our classification criteria. P value was calculated by the log‐rank test among subtypes.


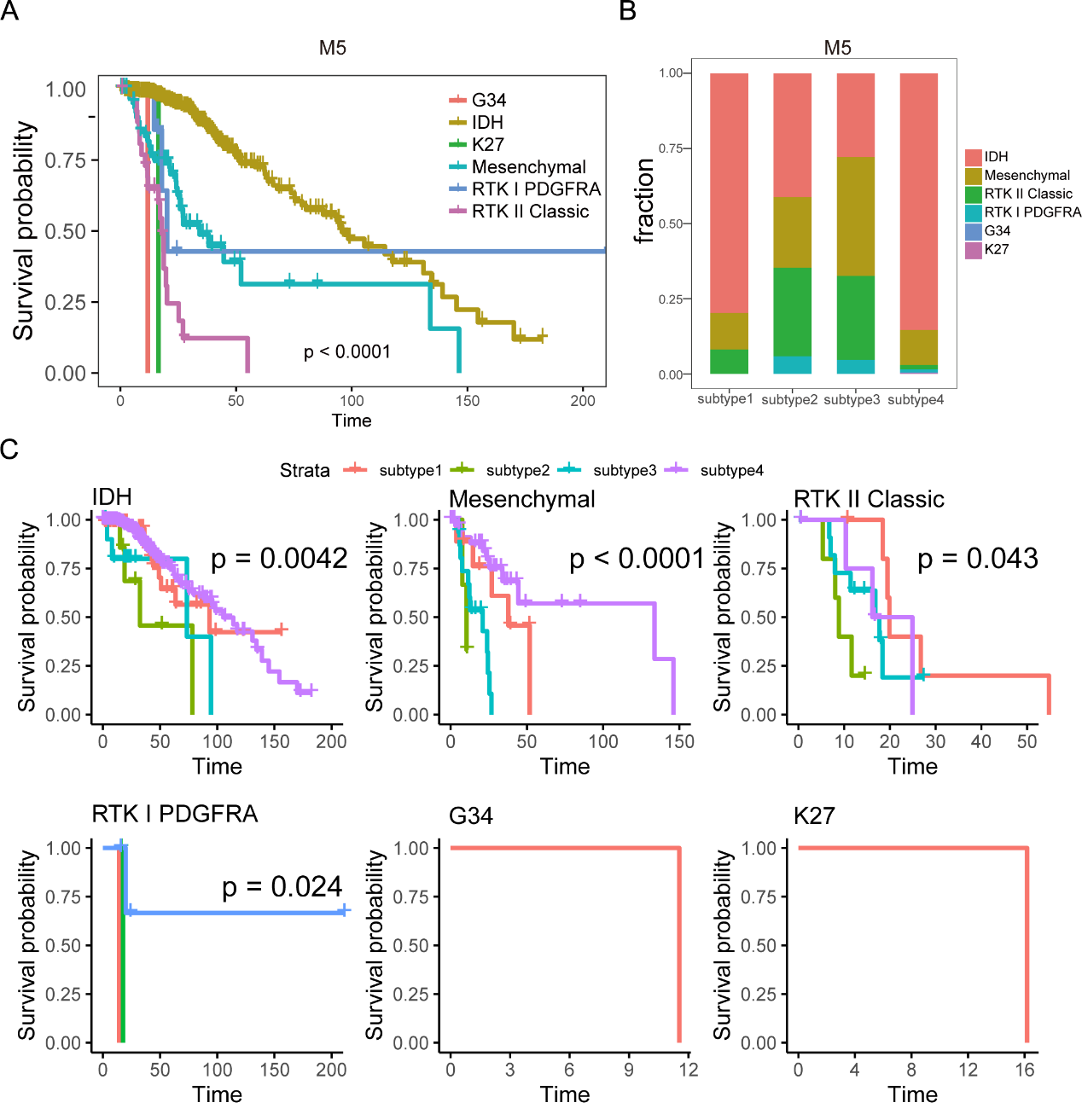


Figure S13. Comparison with other method(M6) **(A)** The Kaplan–Meier analysis of subtypes identified by M7. P value was calculated by the log‐rank test among subtypes; **(B)** Bar plots showed the proportion of tumors stratified by M7 within our subtypes; **(C)** The Kaplan–Meier analysis of each subtype which stratified according to our classification criteria. P value was calculated by the log‐rank test among subtypes.


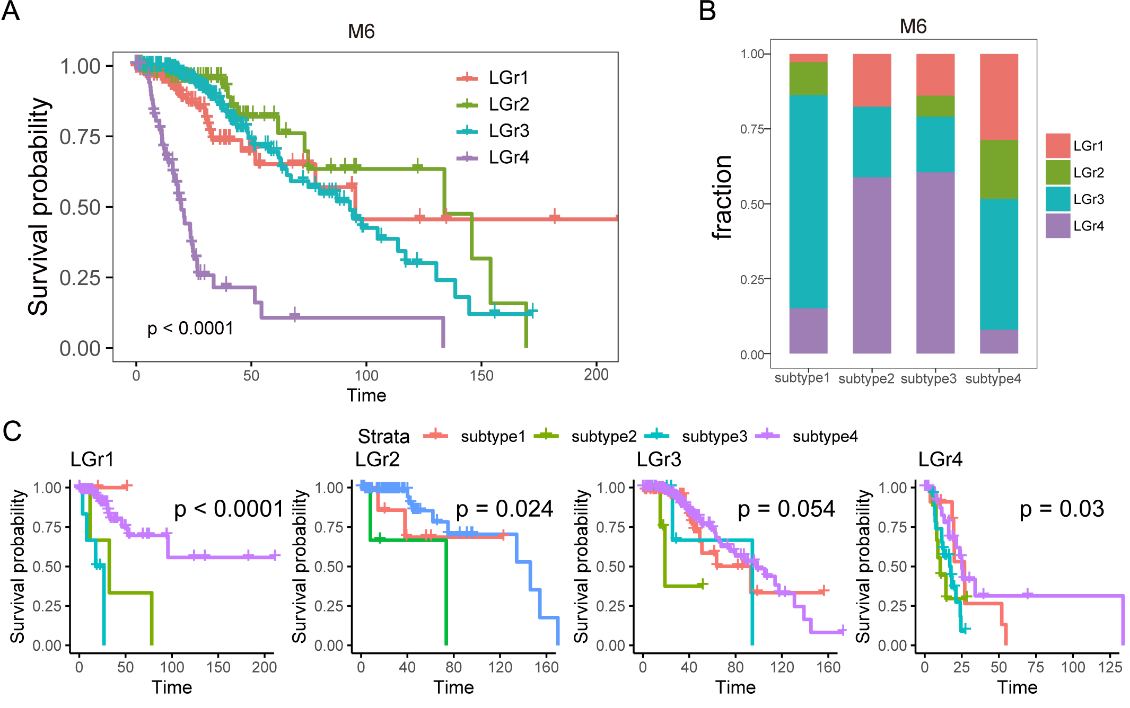


Figure S14. Comparison with other method(M7) **(A)** The Kaplan–Meier analysis of subtypes identified by M7. P value was calculated by the log‐rank test among subtypes; **(B)** Bar plots showed the proportion of tumors stratified by M7 within our subtypes; **(C)** The Kaplan–Meier analysis of each subtype which stratified according to our classification criteria. P value was calculated by the log‐rank test among subtypes.


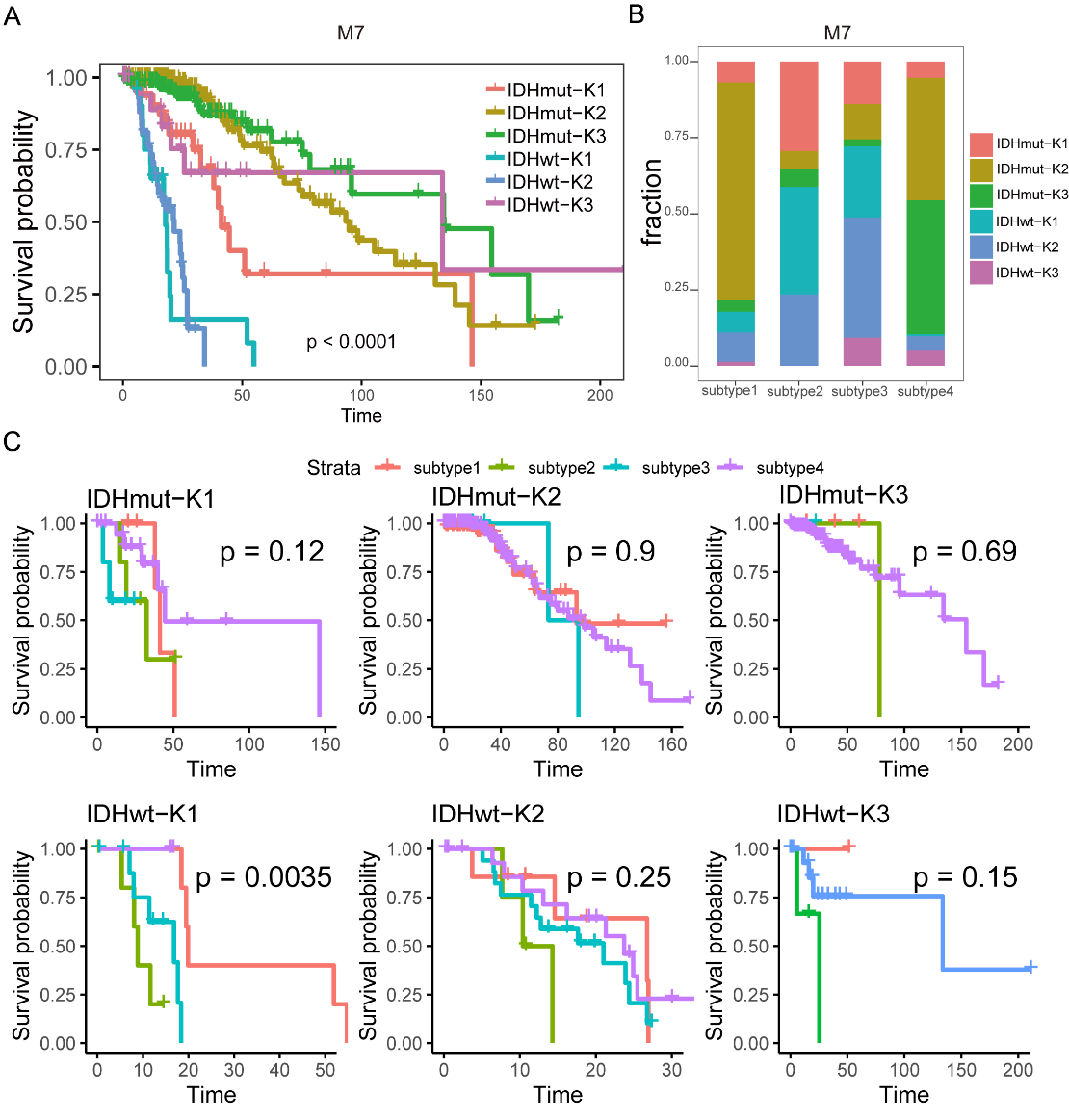


Figure S15. Comparison with other method(M8) **(A)** The Kaplan–Meier analysis of subtypes identified by M8. P value was calculated by the log‐rank test among subtypes; **(B)** Bar plots showed the proportion of tumors stratified by M8 within our subtypes; **(C)** The Kaplan–Meier analysis of each subtype which stratified according to our classification criteria. P value was calculated by the log‐rank test among subtypes.


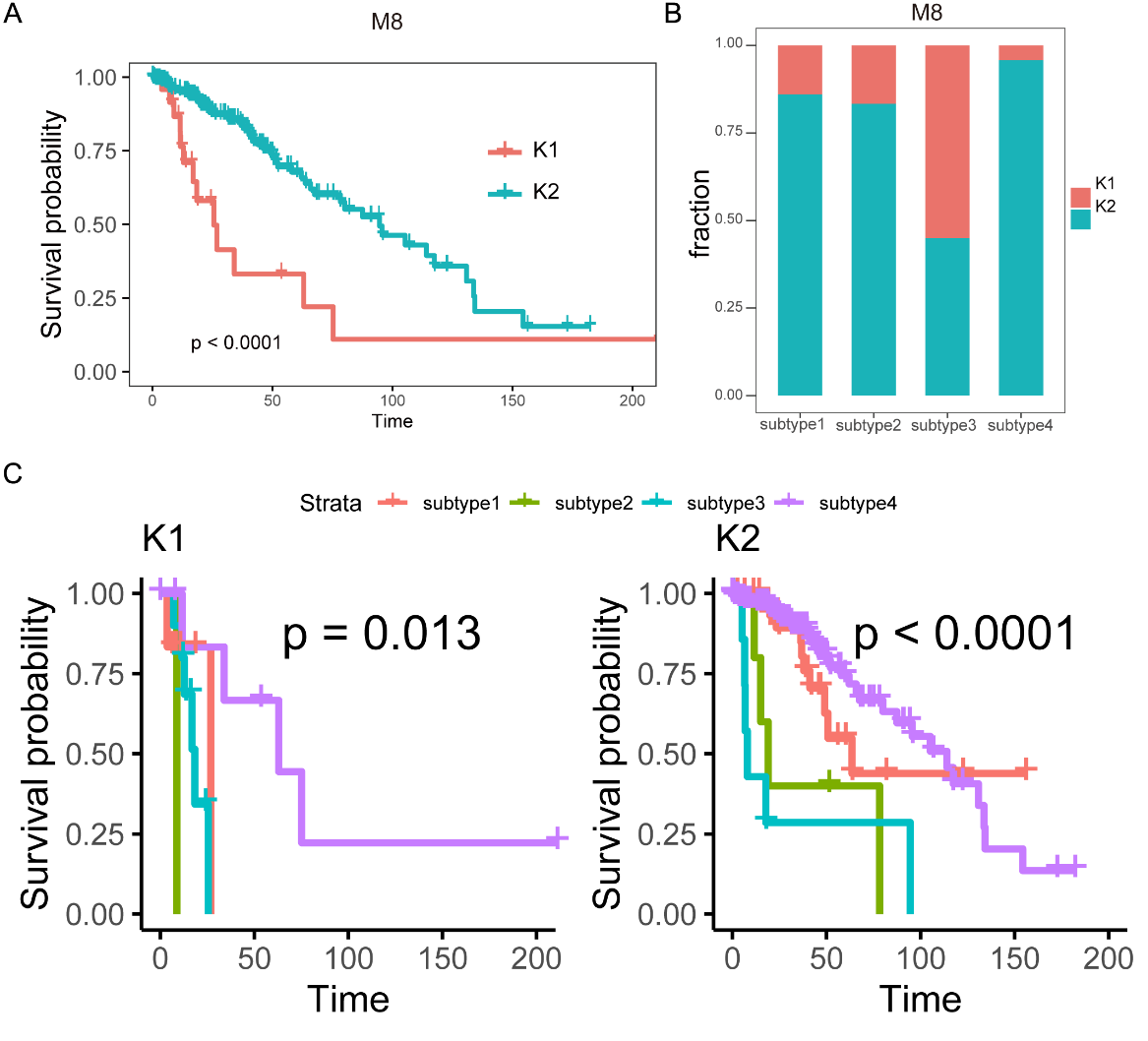


Figure S16. Consensus clustering based on active gene-hallmark pairs of 507 LUAD in TCGA cohort **(A)** The consensus clustering heatmap for LGG; **(B)** The Kaplan–Meier analysis of subtypes identified by our method based on OS. P value was calculated by the log‐rank test among subtypes.


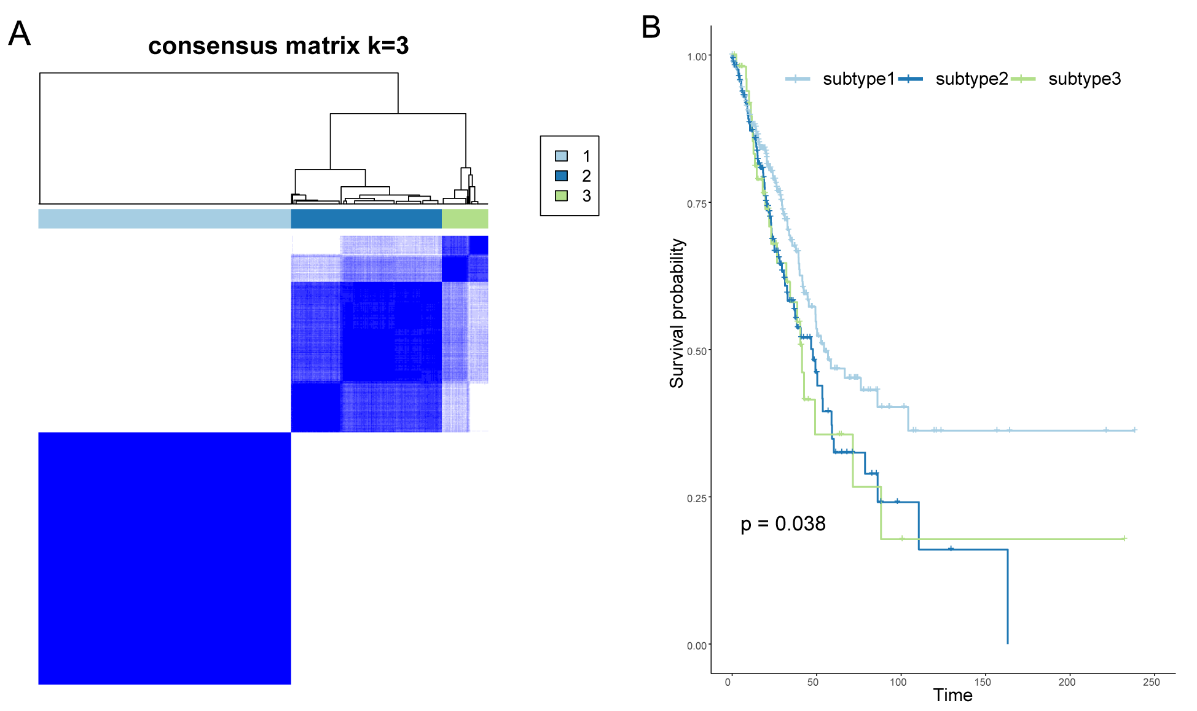


**Supplement Tables**

Table S2. Comparison of subtypes with each other

| **Comparison object** | **P-value** |
| --- | --- |
| subtype4/subtype1 | 0.089 |
| subtype4/subtype2 | 3.07e-13 |
| subtype4/subtype3 | 0.001 |
| subtype1/subtype2/subtype3 | 2.37e-07 |
| subtype1/subtype2 | 1.77e-05 |
| subtype1/subtype3 | 1.64e-07 |
| subtype2/subtype3 | 0.89 |

Table S4. The information of previous method

| **Previous method** | **Main idea** | **Data** | **Limitations** |
| --- | --- | --- | --- |
| Ping et al | This method can characterize lncRNA functions in a direct way based on SCNA-induced dysregulated ceRNA network | miRNA-PG/miRNA-lncRNA target interactions data; copy number data; gene expression data | This method can be affected by the input data. The potential driver SCNAs with low expression levels and SCNA frequencies cannot be identified |
| Zhou et al | The method identifies driver SCNAs (oncogenes or tumor suppressor genes) in genomic regions where abnormalities often occur, including a number of genes or non-coding genes | miRNA-PG/miRNA-lncRNA target interactions data; copy number data; gene expression data; protein interaction data | The driving roles of genes in cancer have not been fully considered and analyzed |
| DriverDBv3 | This method uses published bioinformatics algorithms to identify driver genes | Cancer omics data | It cannot dissect driver roles of genes in cancer in a direct manner |
